# Sleep among Youth with Severely Disabling Chronic Pain: Before, during, and after Inpatient Intensive Interdisciplinary Pain Treatment

**DOI:** 10.3390/children8010042

**Published:** 2021-01-12

**Authors:** Kendra N. Krietsch, Dean W. Beebe, Christopher King, Kendra J. Homan, Sara E. Williams

**Affiliations:** 1 Department of Psychology, 1 Children’s Place Suite 3N14, St. Louis Children’s Hospital, St. Louis, MO 63110, USA; 2 Department of Clinical Pediatrics, Washington University, St. Louis, MO 63130, USA; 3 Division of Behavioral Medicine and Clinical Psychology, Cincinnati Children’s Hospital Medical Center, Cincinnati, OH 45229, USA; dean.beebe@cchmc.org (D.W.B.); christopher.king@cchmc.org (C.K.); kendra.homan@cchmc.org (K.J.H.); sara.williams@cchmc.org (S.E.W.); 4 Department of Pediatrics, University of Cincinnati College of Medicine, Cincinnati, OH 45267, USA; 5 Center for Understanding Pediatric Pain (CUPP), Cincinnati Children’s Hospital Medical Center, Cincinnati, OH 45229, USA

**Keywords:** adolescent, pain rehabilitation, sleep timing, sleep variability, sleep quality

## Abstract

Poor sleep is commonly reported in pediatric chronic pain. There are signals that intensive interdisciplinary pain treatments (IIPT) may inadvertently improve objective sleep, but this claim cannot be substantiated without baseline sleep data prior to IIPT. This study followed the objective sleep/wake patterns (e.g., duration, quality, timing, consistency) of pediatric patients with severely functionally disabling chronic pain before, during, and after inpatient IIPT (the Functional Independence Restoration Program—“FIRST Program”), alongside a similarly-disabled chronic pain Comparison Group. The final sample included *N* = 10 FIRST Patients and *N* = 9 Comparison Group patients. At baseline, the whole sample showed healthy sleep duration (~9 h), average sleep efficiency <90%, late sleep onset and offset (mean = 11:56 p.m.–8:50 a.m.), and highly inconsistent sleep schedules night to night. During IIPT, FIRST Patients maintained healthy sleep durations, moved sleep schedules 2 h earlier, and decreased timing and duration variability by >60 min while the Comparison Group maintained similar sleep to baseline. At follow up (1–2 months later), FIRST Patients’ sleep schedules shifted later but were still less variable than at baseline. Results point to the malleability of sleep/wake patterns within treatment contexts with strict environmental control but suggest that these gains may be difficult for youth with chronic pain to maintain in the home environment.

## 1. Introduction

Pediatric chronic pain (pain lasting 3 months or longer) is an underappreciated and understudied condition [1]. In American samples, prevalence rates of chronic pain are reported to be approximately 8% in typically-developing youth and up to 16% among youth with neurodevelopmental differences, such as autism. Although no American studies document the prevalence of severely disabling chronic pain, approximately 5% of Spanish youth [2] experience chronic pain severe enough to impair broad domains of functioning, including school and academics, socialization, mood, health behaviors, and engagement in pleasurable activities [3,4,5,6,7,8]. These youth often have withdrawn from normative childhood experiences that are central to their biopsychosocial development. For example, youth with severe chronic pain have more school absences and participate less in non-school activities [9]. In addition to being enriching and mastery-building experiences, these types of activities also help serve as cues for other health behaviors, such as sleep. For example, attending school anchors consistent wake times on weekday mornings [10]. Playing in an outdoor recreational basketball league provides daytime light exposure, physical activity, and socialization. Combined with consistent sleep timing, these behaviors help to stabilize the body’s internal timekeeper, the circadian rhythm [11].

### 1.1. Sleep in Pediatric Chronic Pain

Sleep is an important health-related domain that also impacts nearly all realms of functioning. Among healthy populations, inadequate sleep duration and poor sleep quality have been linked to poor academic performance [12], poor mental health [13], and even chronic disease risk via development of obesity [14,15]. There is some evidence that organic sleep concerns are more prevalent among specific pain conditions. For example, higher rates of sleep-disordered breathing are well documented in pediatric sickle cell disease [16,17] and emerging in chronic headache and migraine [18,19,20]. However, a greater number of studies investigate self-reports of sleep and behaviors relating to sleep (e.g., insomnia). Broadly, youth with chronic pain specifically report sleeping less, having poorer sleep quality, and feeling more tired during the day compared to healthy peers [21]. On behavioral measures, youth with chronic pain report experiencing more insomnia-like symptoms, including difficulty falling and staying asleep [22]. There is also evidence that youth with chronic pain engage in more unhealthy pre-bedtime sleep habits, such as spending time in bed during the day, and have higher pre-sleep arousal (e.g., feeling “keyed up”) [23]. The few studies that used objective measures of sleep (such as wrist-mounted actigraphy) suggest similar sleep durations across groups, but youth with chronic pain have more nighttime awakenings versus healthy peers [24].

Findings also show that poor sleep may feed back into the pain experience. Sleep and pain appear to be bi-directionally related—with research showing that poor nighttime sleep predicts worse next-day pain, and daytime pain predicts poorer same-day sleep [25]. It appears that, not only does pain interfere with sleep or habits that impact sleep, but poor sleep makes managing pain more difficult. Studies also suggest that sleep concerns may be particularly relevant to the functioning of youth with chronic pain, with poorer self-reported sleep quality predicting worse health-related quality of life and poorer physical functioning in these youth [26,27].

Interestingly, few pediatric pain studies to date have explored sleep beyond organic disorders or measures of perceived sleep duration, nighttime awakenings, or insomnia symptoms. No study, to our knowledge, has examined aspects of sleep that are more directly under the patient’s control, such as the timing and consistency of when they go to sleep and wake up (e.g., sleep/wake patterns). Although sleep duration and quality have received more attention, sleep timing (e.g., early vs. later) and consistency (e.g., intra-individual variability in sleep timing) are becoming increasingly recognized as potential contributors to poor health outcomes across both clinical and non-clinical populations [28]. Cross-sectionally, youth with a later circadian sleep phase (who trend towards later bedtimes and rise times, especially on non-school nights) tend to have shorter sleep durations [29], struggle more academically [30], engage in more risky behaviors (e.g., cigarette, alcohol, and drug use) [29], use more daily screen time [31], and engage in less healthy weight-related behaviors such as eating and physical activity [32]. More variable sleep patterns night to night have also been linked with more challenging behavioral problems such as inattention [33], aggression [34], and risk taking [35,36], as well as adverse metabolic outcomes [37,38,39]. These are crucial areas to investigate in youth with chronic pain. Sleep is likely not only affected by the experience of pain directly, but also indirectly by reactive pain behaviors, habits, or coping strategies (e.g., sleeping in on mornings with worse pain, napping as a means of escape) that may be modifiable and could serve as behavioral treatment targets.

### 1.2. Interventions for Disabling Pediatric Chronic Pain

There is evidence that many youth with chronic pain benefit from outpatient multidisciplinary intervention [40]. Treatment typically consists of a combination of medication management, psychological treatment (e.g., cognitive behavioral therapy), and physical therapy to address the dynamic interplay of factors (e.g., biological, psychological, sociocultural factors) that contribute to chronic pain [41,42,43]. Overall, treatment from all disciplines is focused on improving function (e.g., walking, attending school or work), which usually improves before patients experience a change in pain [40].

Unfortunately, youth with the highest levels of functional disability and pain may fail to respond to traditional outpatient therapies [44,45,46]. In those cases, intensive interdisciplinary pain treatment (IIPT) programs, either inpatient or day treatment, may be the next step. Like outpatient multidisciplinary interventions, the primary goal of these programs is to increase patients’ ability to function effectively in their world. However, treatment is intensified by structured therapeutic activities in a controlled setting. One of the few pediatric inpatient IIPT programs, the Functional Independence Restoration (FIRST) program, is located at Cincinnati Children’s Hospital on the inpatient rehabilitation unit. Emerging clinical research shows that IIPT is effective at reducing functional disability among most patients [47,48,49], and that those functional improvements sustain over time for many. However, few studies have investigated possible secondary benefits of such programs on other important psychological [50] or sleep-related [51] outcomes that may also have profound impacts on improving functioning and quality of life.

### 1.3. Preliminary Data

To bridge this gap, the FIRST program began tracking patients’ sleep and providing clinical feedback to patients with a wrist-mounted sleep-monitoring device. Preliminary evidence suggests that, within their first week of IIPT, most patients exhibit developmentally appropriate sleep timing, stability, and duration (Boggero et al., under review). While these findings are in line with anecdotal reports of improved sleep in patients with chronic pain attending IIPT, these results contrast with the literature and patient reports of poor self-reported sleep outside of the IIPT setting. However, that preliminary study lacked baseline sleep data and, thus, could not show changes in sleep upon entry into IIPT. Further, it had no comparison group of similar youth receiving only outpatient care. Clinically, it appears that IIPT may indirectly improve sleep in patients with chronic pain. However, to empirically test that impression, sleep data are needed on IIPT patients prior to admission, as well as a comparison to youth with similar levels of pain-related functional impairment.

### 1.4. Study Aims and Hypotheses

The purpose of this study was to replicate preliminary findings and extend our prior work in a new sample of adolescents with severely functionally disabling chronic pain. First, we aimed to describe the objective sleep/wake patterns (e.g., duration, quality, timing, consistency) of youth with severely disabling chronic pain prior to IIPT, building upon the limited existing research by using objective sleep measures. Second, we aimed to evaluate the impact of IIPT on sleep among those receiving treatment (*FIRST Patients)* compared to a similarly disabled sample of pediatric chronic pain patients receiving outpatient treatment as usual (*Comparison Group*) across three time points (Time 1—baseline, prior to IIPT; Time 2—final week of admission to IIPT or the equivalent period of time for the Comparison Group; and Time 3—follow up, approximately 1–2 months later, corresponding to the common timeframe for post-discharge outpatient follow up after the FIRST program). We hypothesized that the FIRST Patients would, from Time 1 (baseline) to Time 2 (final week of IIPT), exhibit earlier sleep timing and decreases in intra-individual sleep timing variability, with slight attenuation in these effects at Time 3 (follow up). In contrast, we expected the Comparison Group to exhibit minimal changes in intra-individual sleep timing and variability across three time points. We anticipated both groups would maintain similar sleep duration and sleep quality over time.

## 2. Materials and Methods

This study was approved and overseen by the Institutional Review Board at Cincinnati Children’s Hospital Medical Center (IRB ID: 2019-0216). Parents provided written informed consent and youth participants provided written assent to participate.

### 2.1. Participants and Recruitment

All patients were recruited from an outpatient pediatric chronic pain clinic at a large Midwestern tertiary children’s hospital (Cincinnati Children’s Hospital Medical Center). The purpose of this pain clinic visit was to evaluate the patient for appropriateness for IIPT. To be clinically considered for admission to the FIRST Program, patients had to have experience moderate to severe functional disability across several domains and failed to progress in traditional outpatient therapies (e.g., physical or aquatic therapy, pain-focused cognitive behavioral therapy). Patients also needed to be medically cleared for physical activity and have completed relevant medical or diagnostic testing.

Staff approached every eligible patient during their pain clinic visit between March 2019 and March 2020. To be eligible to participate in this study, patients needed to be 8–18 years old, have moderate to severe functional disability as a result of their pain, and be evaluated for admission to the FIRST Program within that chronic pain clinic visit (even if they were not ultimately admitted). Exclusion criteria included (1) patient diagnosed with a non-pain-related condition known to have significantly altered sleep (such as autism or bipolar disorder) or known to create barriers to sleep monitoring (such as intellectual disability), and (2) patient and/or family were non-English speaking. Patients who were not ultimately admitted to the FIRST Program due to logistics challenges (e.g., insurance denial) remained eligible for the Comparison Group.

### 2.2. Procedures

Due to ethical concerns about withholding care from vulnerable participants, participants in this study were not randomized by investigators to study group. They were instead followed as part of their clinical care, with some patients being admitted to the FIRST Program (referred to herein as “FIRST Patients”) and some ultimately not being admitted and continuing care as usual (referred to herein as the “Comparison Group”). This was, therefore, a quasi-experimental design comparing FIRST Patients against a Comparison Group with similar levels of pain-related functional impairment, allowing for examination and control as needed for differences in baseline functioning and for the presence and timing of research assessments. Groups were assessed at three points (details on assessment timing are provided in the Results section):

*Time 1* (baseline) occurred directly following outpatient evaluation for the FIRST Program (prior to FIRST admission for the patients who entered the FIRST program). Directly following their pain clinic visit, families were screened, assented, and consented. Study staff provided the family with a watch-like objective sleep monitor and paper-based sleep diaries (both detailed below), as well as a pre-paid envelope to send materials back. Participants were instructed to start wearing the sleep monitor 24 h/day (except bathing or swimming) for the next week, and to complete a brief sleep diary each morning. On the 7th day of Time 1, participants were asked to mail back the sleep tracking materials.

*Time 2* (final IIPT week or equivalent time) occurred during the final week of admission for the FIRST Group. Because FIRST Patients already wear identical sleep trackers as part of clinical care, these data were collected from medical records. Patients in the Comparison Group completed Time 2 during a week yoked to FIRST Patients, based on the median historic span of time between initial evaluation and discharge from the FIRST program, which was updated quarterly. Comparison Group patients were mailed the sleep materials along with a pre-paid return envelope, with instructions identical to Time 1.

*Time 3* (post-discharge follow up or equivalent time) occurred directly following the post-discharge follow-up outpatient visit in the pain clinic for the FIRST Patients. Comparison Group patients completed Time 3 at the median historic time span between FIRST Program discharge and outpatient follow up, updated quarterly. FIRST Patients received their sleep monitors in-person during their pain clinic visit and Comparison Group patients were mailed the monitors. Both groups were supplied with a pre-paid envelope to mail the devices back in. On the 7th day of Time 3, participants mailed back the sleep monitors and diaries.

Following completion of the study, a trained study staffer (Ph.D. level psychology fellow with training in pediatric sleep, first author KNK) sent patients a feedback letter. This was based on that patient’s sleep monitoring data and other subjective sleep data not reported in this manuscript, compared against developmental recommendations for sleep. When there were concerns for clinically significant sleep problems, patients were provided with contact information for pediatric sleep providers and encouraged to pursue clinical follow up.

### 2.3. Inpatient Pain Rehabilitation Setting

The FIRST Program is an interdisciplinary inpatient pain rehabilitation program that focuses on restoring function in patients who are severely impaired by chronic pain. Patients are admitted to the inpatient rehabilitation unit, where they are followed daily by rehabilitation and pain physicians, pain psychologists, physical therapists, occupational therapists, nurses, nurse practitioners, social workers, school services, child life specialists, music therapists, massage therapists, and recreational therapists. Weekdays are highly structured, with patients attending 5–6 h of individual therapy sessions in addition to at least an hour of school. Patients have formal physical and occupational therapies on Saturday morning as well. Therapies share the same overall treatment goal: for the patient to return to previous functional levels of physical activity, become more independent, and learn effective ways to cope with distress and pain. In physical therapy, patients take a graded approach to building muscle strength, stamina, coordination, and balance. Occupational therapy focuses on building independence in completing activities of daily living related to academics, self-care, and leisure. In psychology, patients learn about the mind/body connection and strategies to improve self-management of their pain, including behavioral (e.g., deep breathing, muscle relaxation, biofeedback) and cognitive techniques (e.g., identifying thinking patterns that amplify the pain experience, goal setting).

Patients also follow a structured schedule in their independent time in order to stay on track with daily activities such as mealtimes, independent exercise, and bed/wake times. As part of the FIRST program rules, patients are expected to have lights out and technology (including TV and phones) turned off by 10:00 p.m. Patients must wake by 7:15 a.m., in time to prepare for 8:15 a.m. therapies. Lights and blinds must stay on and open during the day, and patients are not allowed to nap throughout the day. Although most of the FIRST program was not specifically designed to address sleep concerns, patients also receive weekly feedback about their sleep monitor from the program psychologist (anchor author, SEW) as part of their standard clinical care. Program length is individualized to the patient and in this sample was 20 days, on average (range = 11–29 days). Prior research has shown efficacy of this treatment approach in improving functioning in patients with previously severe functional disability [49].

### 2.4. Measures

*Demographics.* For this study, the following demographic variables from the initial pain clinic visit were abstracted from the medical record: age, sex, race, ethnicity, insurance type (commercial or Medicaid), and spatial distribution of pain (localized vs. widespread), which was based initial pain clinic diagnosis. Localized pain included pain diagnosis/diagnoses pertained to ≤2 specific body parts (e.g., abdominal pain, headache, back pain, knee pain, CRPS of 1 limb) while widespread pain included diagnosis reflective of multiple body parts affected (e.g., fibromyalgia, generalized pain, EDS). For patients with multiple pain types, the most functionally disabling pain type was categorized as primary. For Comparison Group participants, reason for not being admitted to the FIRST Program was obtained from documentation in the patient’s medical record and confirmed with the FIRST Program coordinator.

*Functional Disability Inventory (FDI):* Patients completed the FDI as part of their clinical care at their Pain Clinic Visit at Time 1 (baseline); scores were extracted from the medical record to characterize and compare levels of disability across the FIRST and Comparison Groups. The FDI is a 15-item self-report inventory assessing youth’s perceived difficulty in the performance of daily activities in home, school, recreational, and social domains (Walker & Greene, 1991). The FDI is a valid, reliable measure of functional ability that has been clinically normed and is widely used in pediatric chronic pain populations as a primary clinical treatment outcome measure [52]. Youth rate the degree of difficulty of daily tasks due to physical health problems on a 5-point Likert scale ranging from 0 “no trouble” to 4 “impossible,” with higher scores representing more disability. Internal consistency (reliability) was high in this study, α = 0.89.

*School Attendance.* Patients were asked at all three time points about their schooling during the monitoring week. Questions included whether youth were attending school that week, school context (e.g., traditional brick-and-mortar school, online school, homebound schooling with a visiting teacher, homeschooling without enrollment in formal education), and whether schooling was structured (e.g., required to attend or login to classes at scheduled times of day) or unstructured (e.g., could be completed at any time of day).

*COVID-19 Shutdown.* Although most data were collected prior to the societal disruption due to COVID-19, some were not. Time points that were completed prior to 17 March 2020 (the date that in-person school attendance was shut down in the state of Ohio due to declaration of state of emergency) were coded as “pre-shutdown” and time points that were completed after 17 March 2020 were coded as “during shutdown”. Data collection for this study stopped prior to reopening of in-person schooling.

**Figure 1:**
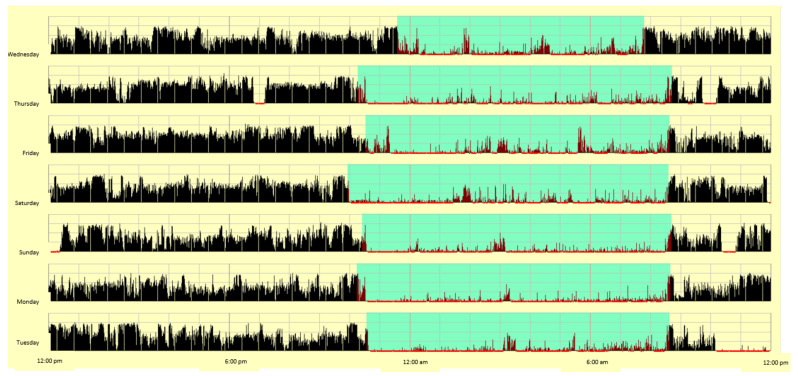
Visual histogram derived from sleep monitor.

*Objective Measure of Sleep/Wake Patterns.* Patients wore the Motionlogger Sleep Watch (Ambulatory Monitoring, Ardsley, NY, USA), which provides continuous monitoring of gross motor activity to produce objective, algorithm-derived indices of sleep. The Motionlogger Sleep Watch is worn on the non-dominant wrist and is generally well tolerated in pediatric populations. Patients were instructed to wear the device 24 h/day, as well as complete a brief morning sleep diary, for 7 nights at each of the 3 time points. Staff used questions from the sleep diary about time into and out of bed to screen for artifacts (e.g., device malfunction or removal) and to set plausible sleep periods within which to apply the scoring algorithm. All data were scored with the widely-used “Sadeh” algorithm, which has been validated in healthy 3–18 year olds and yields sleep/wake estimates that are >90% concordance with polysomnography [53]. As a safeguard against possible implausible algorithm-derived sleep indices, staff visually cross-referenced each individual night of quantitative sleep data with the software program’s histogram (graphical display of nightly data). This was helpful for detecting the rare occasion when sleep timing data were inconsistent with that unique patient’s general sleep/wake pattern, and may have been the result of a data processing error. See Figure 1 for an example of the software’s visual histogram of 1 week of sleep data. To be included in primary analyses, patients needed data on at least 4 nights, including at least 1 weekend night. As noted below, we also post-hoc explored whether primary analyses replicated when examining weeknights only for participants with data from at least 3 such nights.

**Sleep timing** was assessed via indexes of (a) *sleep onset*, as indexed by the first clock time of consistent algorithm-scored sleep, averaged across days within each of the three measurement periods; and (b) *sleep offset*, the clock time of last awakening for the day, averaged across days within each measurement period.

**Sleep duration** was assessed via the *sleep period*, or the simple difference between sleep onset and sleep offset, averaged across days within each measurement period; higher values indicate a longer period of sleep.

**Night-to-night variability in sleep** was measured (with higher scores indicating greater variability) via (a) *sleep onset variability*, computed as intra-individual standard deviation of the time of sleep onset, across days within each time point, in minutes; (b) *sleep offset variability*, the intra-individual standard deviation of the time of sleep offset across those days, in minutes; and (c) *sleep period variability*, the intra-individual standard deviation of sleep period across those days, in minutes.

**Sleep quality** was measured via *sleep efficiency*, reflecting the percentage of the sleep period actually spent in sleep across the days within each measurement period; higher values indicate better quality sleep.

### 2.5. Analytic Approach

In order to run analyses (which require continuous numeric values), sleep onset and sleep offset clock time were converted into minutes past 12:00 h. Numeric values were converted back into clock times when presented in descriptive statistics and graphs. Descriptive background statistics including the mean, standard deviation, and frequencies were calculated at Time 1(baseline). The FIRST Patients and Comparison Group were then compared on these variables at baseline using independent samples t-tests or chi-square, as appropriate. We tested ANOVA assumptions via the Shapiro-Wilk index to assess the normality of distributions, Levene’s test to assess homogeneity of variance, and Mauchly’s test to assess sphericity of repeated measures. To test whether sleep differed by group across time, we conducted a 3 (time) by 2 (group) mixed ANOVA with an alpha of 0.05. This model was used due to its robustness against violations of assumptions that can arise in small samples [54]. This analysis also allowed for examination of group by time interactions on timing and variability in sleep onset and offset, duration and variability in sleep duration, and sleep efficiency. Follow-up post-hoc tests for group differences in sleep at Time 1 (baseline) were conducted using independent samples t-tests. Time 2 (final IIPT week or equivalent time) and Time 3 (post-discharge follow up or equivalent time) post-hoc tests were conducted using ANCOVA in order to control for Time 1 (baseline) sleep. Given the large number of post-hoc contrasts, a Bonferroni correction of alpha = 0.01 (0.05/5 unique outcomes) was used to protect against Type 1 error. Finally, as exploratory sensitivity analyses, we post-hoc repeated our primary ANOVA to explore group by time effects (a) after removing potential outliers, (b) after removing potential outliers and log transforming the data to address violations of normality, (c) using non-parametric Mann–Whitney U tests to compare groups in changes in sleep from Time 1 to Time 2, as well as Time 1 to Time 3 sleep, and (d) examining sleep onset, offset, duration, and efficiency on *weeknights* only. These sensitivity analyses allowed us to examine whether effects shown in the primary analyses could be readily attributed to distributional issues affecting analytic assumptions (sensitivity analyses a–c) or by specific portions of the week (sensitivity analysis d). Outcome variables were non-normally distributed and/or showed heterogeneity of variance across selected time points and sleep outcomes, but nearly all of these were normalized after log transforming the data and removing outliers (See Supplementary Table S2), so those data were used to conduct sensitivity analyses. There were no violations of sphericity via Mauchly’s test.

For all analyses, partial eta squared (η_p_^2^) effect sizes were presented as appropriate and interpreted as either a small (η_p_^2^ = 0.01), medium (η_p_^2^ = 0.06), or large (η_p_^2^ = 0.14) effect size [55].

## 3. Results

Approximately 50 patients were considered for admission to the FIRST Program, of which 36 consented into the study. Primary reasons for not enrolling in the study included lack of interest and being admitted to the FIRST Program too soon after evaluation to participate in Time 1 baseline sleep monitoring. Of the 36 consenters, 32 completed Time 1 (baseline) data collection (4 dropouts: 2 failed to return materials and 2 withdrew participation after the first night). Of those, 29 (FIRST Group *N* = 17, Comparison Group *N* = 12) completed Time 2 (final IIPT week or equivalent time) data collection (3 dropouts: 2 failed to respond to communication attempts and 1 withdrew due to declining medical status). Of those, 26 (FIRST Group *N* = 15, Comparison Group *N* = 11) completed Time 3 (post-discharge follow up or equivalent time) data collection (1 underwent psychiatric hospitalization and 2 were lost to follow up). Of the 26 Time 3 completers, 19 met the final data inclusion criteria, which consisted of having ≥4 nights of sleep data with at least 1 weekend. Most dropouts were due to sleep monitor malfunctions resulting in insufficient data. Among the Comparison Group, primary reasons for not being admitted to the FIRST Program included needing to complete medical testing and outpatient therapies, needing to stabilize mood or enhance motivation to complete the program, and insurance barriers. The final sample included 10 patients from the FIRST group and 9 patients from the Comparison Group.

See Table 1 for descriptive information. Across both groups, patients were primarily adolescent, female, Caucasian, with localized or widespread musculoskeletal pain, and covered by commercial insurance. This is similar to previous reports of pediatric chronic pain patients with high levels of pain-related disability [49]. The groups did not significantly differ in age, sex, race, pain localization, insurance coverage, severity of functional disability, season recruited, or percent of visits completed relative to the COVID-19-related shutdown (*p* > 0.05). There was a significantly shorter amount of time between Time 1 (baseline) and Time 2 (final IIPT week or equivalent time) amongst the FIRST Patients. Distribution of schooling also differed across groups over time. Although approximately 1/3 of both groups were attending brick-and-mortar schools at Time 1 (baseline), this rose to nearly ¾ by Time 3 (post-discharge follow up or equivalent time) in the FIRST group, at which point attendance dropped to ~1/5 for the Comparison group.

Table 2 summarizes primary sleep outcomes for patients with complete data across all three time points for both groups. Baseline sleep/wake patterns during Time 1 (baseline) were similar between the FIRST Patients and Comparison Group. Both groups averaged sleep onset near midnight and offset approximately 9 h later, which meets American Academy of Sleep Medicine consensus guidelines for recommended 8–10 h of sleep/night for adolescents [56]. Across groups, sleep timing was quite variable, reflecting an intra-individual standard deviation of 60–90 min in sleep onset, offset, and duration night to night at Time 1 (baseline).

**Figure 2:**
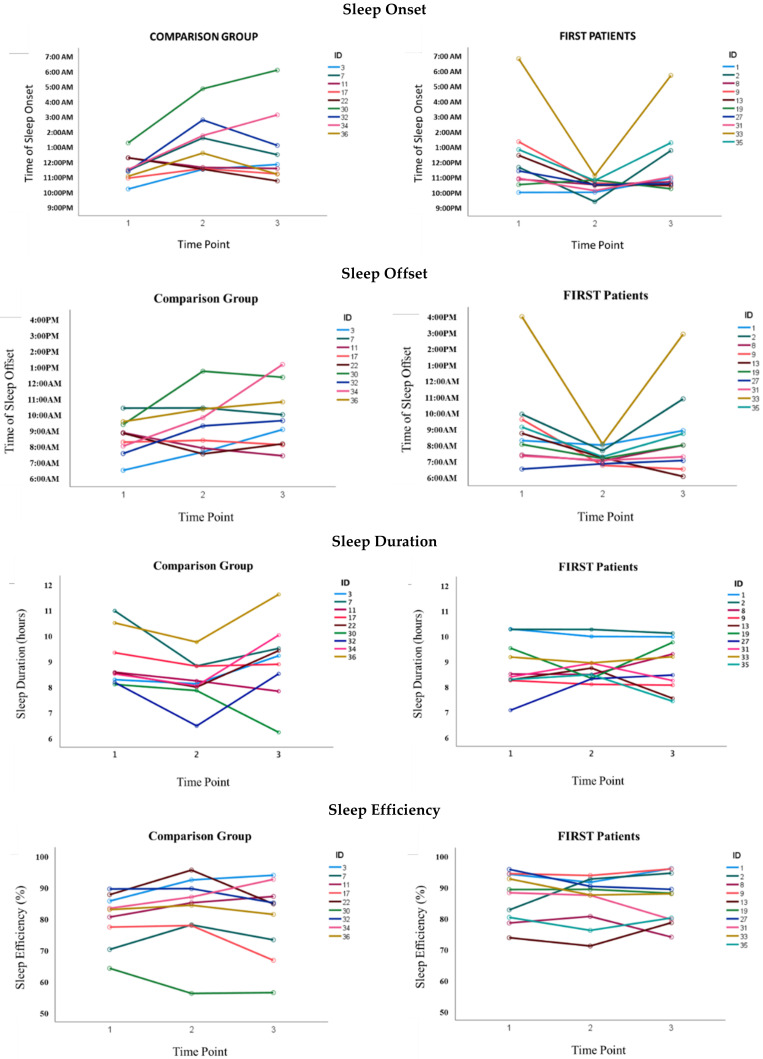

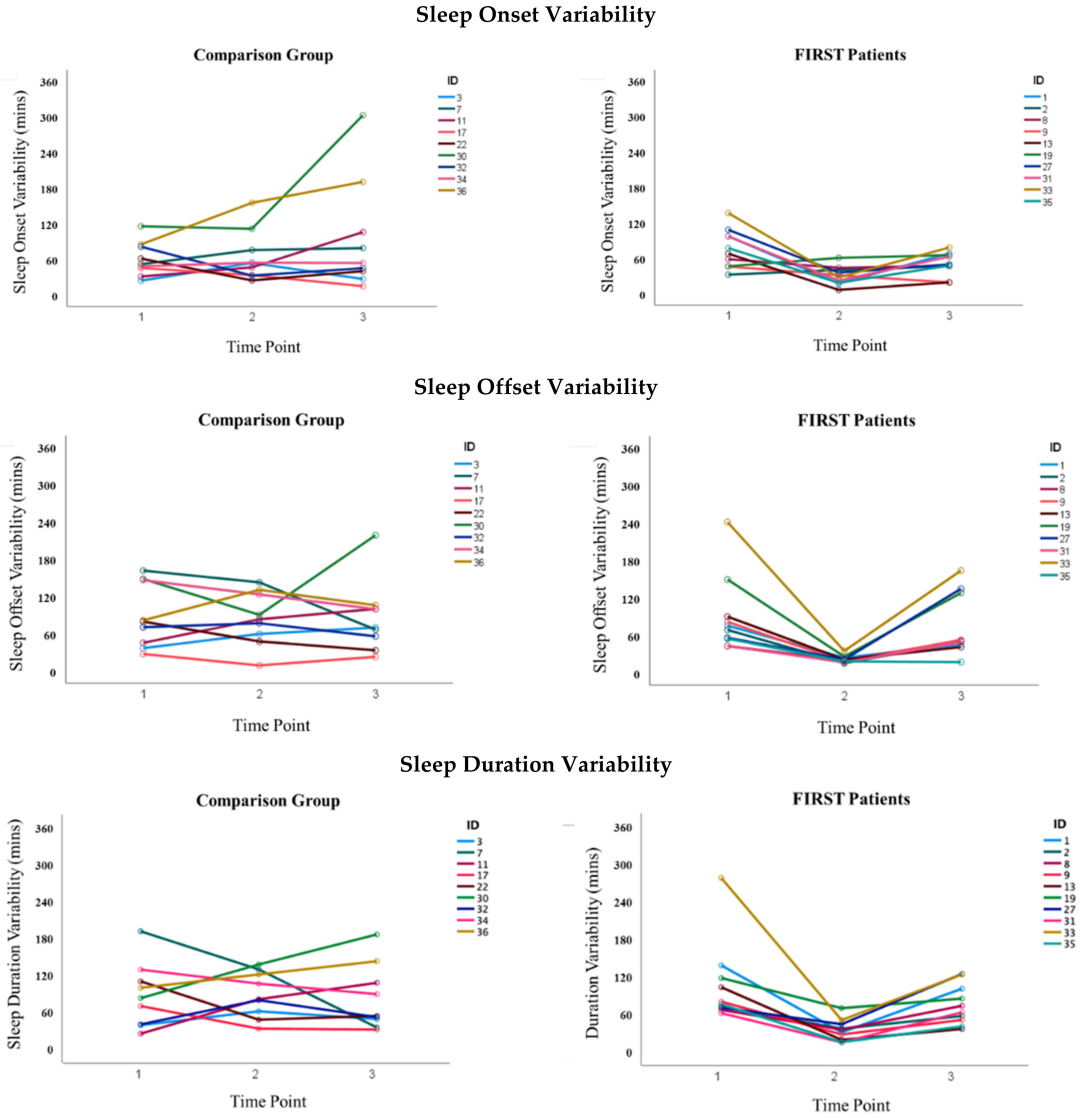
Graphs of estimated means across time points for all sleep outcomes by group. Notes: FIRST Patients = patients receiving IIPT through the Functional Independence Restoration (FIRST) Program; Comparison Group = similarly disabled sample receiving outpatient treatment as usual. Time points corresponded to before (baseline, Time 1), during (final IIPT week or equivalent time, Time 2), and following (post-discharge follow up or equivalent time, Time 3) inpatient IIPT.

See Table 3 for results from the mixed ANOVA and Figure 2 for graphs of all primary sleep outcomes. There were significant group by time interactions for sleep onset timing (*p* = 0.001) and variability (*p* = 0.02), sleep offset timing (*p* = 0.02) and variability (*p* = 0.02), and sleep duration variability (*p* = 0.02); all differed between the FIRST Patients and Comparison Groups across time points. There were no significant effects for sleep duration and sleep efficiency. Exploratory sensitivity analyses essentially replicated these findings when (a) removing outliers unique to each outcome (identified using box-and-whisker plots; see online Supplementary Table S1), (b) when using log-transformed data and removing outliers to address violations of normality (see online Supplementary Table S2), and (c) when examining data on weeknights only (see online Supplementary Table S3). This bolstered confidence in results from our primary analyses.

Primary post-hoc analyses, summarized in Table 4, then probed cross-group differences over time. At Time 1 (baseline), the FIRST and Comparison Groups did not differ on any outcome variable (*p* > 0.05). Post-hoc ANCOVAs revealed that the strongest group effects, covarying for Time 1 (baseline), were found at Time 2 (while the FIRST Patients were actively receiving inpatient pain rehabilitation). Group membership explained 40–60% of the variance in most sleep outcomes. Compared to their counterparts receiving care as usual, while receiving inpatient pain rehabilitation, FIRST Patients fell asleep 2.5 h earlier, woke 2 h earlier, decreased their sleep onset and sleep offset variability to only 20–30 min (vs. 65–85 min in the Comparison Group), decreased sleep duration variability by over 60 min, and maintained a sleep duration of approximately 9 h. At Time 3 (post-discharge follow up), FIRST Patients’ sleep onset and offset timing became later and looked like their Time 1 (baseline) sleep timing. They did, however, maintain some gains in staying consistent in their night-to-night sleep onset, offset, and duration variability (see Figure 1). Although not statistically significant, results suggest that the FIRST Patients may have continued to have meaningful differences in sleep onset, sleep offset, and sleep onset variability at Time 3 (post-discharge follow up or equivalent time) compared to the Comparison Group, but effects were obscured by low power.

Exploratory post-hoc sensitivity analyses using non-parametric Mann-Whitney U tests with outliers removed found a similar pattern of results in changes from Time 1 to Time 2 (see online Supplementary Table S4). Compared to the Comparison Group, FIRST Patients showed larger changes towards earlier time of sleep onset and offset from Time 1 to Time 2, as well as greater reductions in variability of sleep onset, offset, and trends towards greater reductions in sleep duration variability. However, when looking at change scores from Time 1 to Time 3, groups did not vary in changes from Time 1 to Time 3 in any of the sleep outcomes.

## 4. Discussion

### 4.1. Overview of Findings

This longitudinal, quasi-experimental study of adolescents with severely disabling chronic pain supported clinical observations that IIPT, while focused primarily on improving waking functioning in youth with disabling chronic pain, markedly stabilized and shifted sleep/wake patterns earlier. Previous work by our group had found that patients receiving IIPT showed consistent, developmentally appropriate sleep duration and timing (Boggero et al., under review). Other studies have also reported positive effects of IIPT on subjective reports of sleep habits [51]. However, this is the first study to (1) document objective sleep/wake patterns in a severely disabled chronic pain population before treatment, (2) measure change in the timing and consistency of sleep/wake patterns during IIPT and at follow up to investigate maintenance of gains in the home environment, and (3) compare these patterns to a similarly-disabled group with chronic pain.

Aligning with previous work measuring objective (but not subjective) sleep duration in pediatric chronic pain populations [24], both the FIRST Patients and Comparison Group achieved a healthy sleep duration at baseline. In fact, adolescents in our sample slept longer than the typical American teenagers—averaging approximately 9 h/night across nights compared to under 7 h/night [57]. However, patients in our sample also exhibited other concerning, but often overlooked, characteristics of sleep. Prior to IIPT, patients averaged a sleep schedule approximating midnight to 9:00 a.m., although their schedules were variable night to night. This late timing likely reflects the preferred sleep schedule of many adolescents [58]. However, such late sleep schedules likely contributed to adolescents’ disability—as it may have prevented them from engaging in functional activities requiring earlier wake times, such as school attendance and sports practice. Although there are currently no clinical guidelines around sleep consistency/variability, our sample evidenced levels of variability in sleep timing and duration that were 1.5–2 times greater than other teen samples with [59] and without [60,61] identified sleep complaints, using the same actigraphy-derived measure of variability (the intra-individual standard deviation). Sleep quality, measured by sleep efficiency (the percentage of time the spent asleep between sleep onset and offset), was also of concern. Most of our sample had low sleep efficiency of <90%, with several patients at 75% or lower. This is also consistent with literature showing that youth with chronic pain tend to have more awakenings and less efficient sleep than healthy peers [24,62,63].

By their final week of treatment in IIPT, our small sample of FIRST Patients evidenced impressive shifts in all measures of sleep timing and variability. FIRST Patients fell asleep and woke up nearly 2 h earlier, generally sleeping from 10:30 p.m. to 7:15 a.m. They also became more consistent in the times they fell asleep, woke up, and how long they slept—reducing night-to-night variability by over 1 h in most cases. In contrast, their peers in the Comparison Group (who completed data collection at home at a similarly timed interval while receiving care as usual) generally maintained late and variable sleep/wake schedules. Some shifted towards even later bedtimes and slightly shorter (but still healthy) sleep duration. When directly comparing the groups, FIRST Patients fell asleep and woke up nearly 2.5 h earlier, and were 3–4 times more consistent in how long they slept and the time they fell asleep and woke up—all while maintaining a healthy sleep duration of 9 h/night. As expected, sleep duration and sleep efficiency were minimally affected in either group.

At follow up (1–2 months after discharge), FIRST Patients maintained some sleep gains, but looked more like the Comparison Group than during IIPT. Except for one outlier with extremely late sleep timing at both baseline and follow up, most FIRST Patients fell asleep between 10:00 and 11:00 p.m. and woke between 6:00 and 9:00 a.m. (approximately 30 min earlier than at baseline). Notably, Comparison Group patients’ wake times largely got later at follow up. Although many FIRST Patients’ sleep variability increased from treatment to follow up, it was almost uniformly lower than baseline, while the Comparison Group had minimal changes over the 3 time points.

### 4.2. Possible Drivers of Sleep Pattern Improvements

Findings in this study naturally raise questions about drivers of sleep improvement among the FIRST Patients, particularly while they were receiving treatment. Previous work on an earlier sample of FIRST Patients [49] suggests that improved sleep may not be due to improved pain. That prior work found that, although patients’ functioning improves very quickly, pain levels often do not decrease until later in the program or post-discharge. Contributors to the powerful effect on sleep may instead include a combination of environmental, behavioral, and motivational factors. The inpatient environment offered strict control over the sleep environment and various factors that are known to stabilize the sleep/wake cycle [64]. Sleep expectations were clearly outlined to patients upon admission, and unit staff members enforced these rules throughout patients’ stay. Unit lights off occurred at 10:00 p.m., patients were expected to be up in time for scheduled therapies at 8:15 a.m., and there were no TV or electronics allowed during the night. From a circadian perspective, patients had exposure to the most powerful factors that entrain the sleep/wake cycle (also called “zeitgebers”). In addition, indoors, morning and daytime light were maximized (patients were always required to have a light on in the room during the day), whereas nighttime light was minimized. Patients received significant daytime physical activity, regularly timed meals, and social interaction with various providers and other patients on the unit.

Patients in the FIRST Program also received weekly, individualized sleep feedback (based on their sleep monitor findings) from the program psychologist. This was accomplished via color printouts of their sleep/wake patterns from the sleep monitors, which allowed patients to visually track their sleep patterns over the course of treatment. Although not directly measured, this may have had a therapeutic effect on sleep by promoting self-monitoring and self-accountability of sleep schedules with the support of objective feedback [65]. While admitted, patients were reinforced and applauded for staying functional and following their prescribed sleep/wake schedule, which potentially impacted sleep self-efficacy and motivation.

Perhaps most importantly, all activities were highly structured and occurred at the same time every day—likely working to stabilize the sleep/wake rhythm. Although this study was not designed to parse out the multiple aspects of FIRST that could have affected sleep, we speculate that the program’s exceptional level of structure around bedtimes and rise times was particularly powerful; sleep changes were evident almost immediately after admission (Boggero, under review), far sooner than would be expected from changes in circadian rhythm. Given that, in healthy youth, parents’ enforcement of regular bedtimes significantly increases the odds of children meeting sleep duration recommendation (likely by achieving an earlier sleep onset) [66], future studies in pediatric chronic pain should investigate parenting practices around enforcement of bed and wake times and sleep hygiene behaviors, such as keeping devices outside the bedroom. This may be particularly relevant in youth with severely functionally disabling chronic pain; only 25% of adolescents in our sample attended structured schooling that required them to wake up at a regular time each day at baseline (pre-treatment).

### 4.3. Sleep Quality

It is worth noting that although this study focused on sleep/wake patterns (and not sleep disorders), patients in this study showed persistently low sleep efficiency (a marker of poor sleep quality). Broadly, sleep efficiency reflects number and duration of nighttime awakenings, which can be due to a variety of biological and behavioral factors. Scheduling too much time to sleep can cause unwanted awakenings [67], although most of the patients in this study were achieving recommended sleep duration. Additionally, organic sleep concerns, sleep-disordered breathing and limb movements, known to cause awakenings and lower sleep quality, may occur at higher base rates in youth with chronic pain [68,69] and were not directly measured in this study. Finally, while not yet examined in adolescents, adults with chronic pain have been shown to have altered sleep architecture with more frequent micro-awakenings (e.g., alpha intrusions) as compared to healthy controls [70] and this could also impact sleep efficiency. At the conclusion of our study, we ultimately referred nearly 75% of the sample either to be assessed by a sleep physician or behavioral sleep medicine provider. This was primarily due to persistently low sleep efficiency scores, despite healthy sleep/wake scheduling, as well as subjective complaints about sleep quality (despite healthy sleep/wake scheduling). It is important to continue to study the interaction of possible organic sleep disorders with modifiable aspects of sleep such as the sleep/wake schedule within the chronic pain population, as their sleep concerns are likely bio-behavioral in nature.

### 4.4. Limitations, Strengths, and Future Directions

Results of this study should be considered in the context of several limitations, most notable being the small sample. Beyond reducing statistical power and limiting our ability to apply strict control against Type 1 error, we were unable to answer many interesting questions about the impact of sleep improvements on other variables of interest, such as disability and pain levels. With the relatively small number of patients admitted to IIPT at a given site in a given year, future studies will need to be multi-institutional and/or use much longer data collection periods to obtain larger samples. Current findings, though preliminary, provide important justification for such expensive and extensive larger-scale work. In addition to increasing statistical power, larger, multisite samples introduce greater analytic flexibility to accommodate issues with non-normal data; although sensitivity analyses offered reassurance that our primary findings were not spurious, an ideal approach would be to use more sophisticated statistical models possible in larger samples. Larger samples could also investigate similarities and differences in sleep by clinical pain profile (e.g., widespread musculoskeletal pain vs. headache), and whether pain profile affects the impact of IIPT on sleep complaints. As it relates to the current population of patients eligible for the FIRST program, differences in clinical outcomes have been reported in patients with localized vs. widespread pain based on diagnosis of a single vs. multiple conditions (including widespread MSK pain) [49]. So, it is possible that these groups also differ in terms of sleep. However, a larger sample is needed to explore this question.

This is of importance, as etiology (and subsequent effective treatment) of sleep problems may vary by pain complaint—although it is notable that all FIRST Patients showed improvements in their sleep/wake patterns during treatment, regardless of pain location. Furthermore, future studies should strongly consider screening for organic sleep concerns, such as sleep-disordered breathing or limb movements, given the high proportion of patients from our study that were referred on to have a formal evaluation by a sleep physician.

Although severely impaired youth are an important, understudied, and vulnerable population, results from this study of such youth may not translate to other pain rehabilitation models (particularly outside the inpatient setting), less disabled pain populations, or pain samples with a greater proportion of males. Although there are no studies examining sex differences in response to sleep interventions, sleep concerns in adolescence tend to be more prevalent and severe among females (vs. males), and females may have more “room to improve” their sleep while in IIPT [71].

Additionally, although actigraphy offers perhaps the best objective way to measure sleep in youth across protracted timeframes, ultimately it uses algorithms to differentiate sleep from wake based on movement patterns, and algorithms that have been validated in healthy youth may not be as well suited for this population. This study found irregular sleep/wake patterns in youth with severely disabling chronic pain, and other studies find high rates of highly sedentary behavior in this population [72], which can be difficult to differentiate from sleep. Future validation studies comparing actigraphy with polysomnography in adolescent chronic pain samples will be important for ongoing work.

Notable strengths of this study include use of objective sleep monitoring and use of a Comparison Group that was very similar in presenting complaint, demographics, functional disability, and sleep features at the time of recruitment.

## 5. Conclusions

This study extends the existing pediatric sleep/pain literature (which has previously focused on subjective sleep complaints, nighttime awakenings, and insomnia symptoms) by focusing on objective sleep/wake patterns and sleep variability, which may have a stronger impact considering changes in sleep preferences and time commitments in developing teenagers. We are the first to document late sleep timing and variable sleep/wake patterns among a small pediatric sample of patients with severely disabling chronic pain. Perhaps more importantly, we showed that in this sample, sleep/wake patterns were malleable—albeit through an intensive inpatient pain treatment program with near complete control over the sleep environment. We also showed that when the supports and structure of the FIRST Program were removed and youth returned home, they struggled to maintain their sleep gains. This makes maintenance of sleep routines an important area for future study, particularly in terms of determining whether a return to poor sleep practices potentially correlates with poor long-term pain and disability outcomes. Improving sleep timing and consistency is particularly relevant for the chronic pain population, given that many of these patients struggle with engaging in functional activities that require them to be physically present at regularly scheduled times of day (e.g., school). Delayed sleep schedules can be yet another point of friction between coping with pain from a withdrawn standpoint (e.g., staying home to sleep in after a poor night’s sleep) and taking a more functional approach to managing pain.

When comparing the impact of this intervention on sleep timing and consistency, the results are impressive. Where patients in this study decreased intra-individual variability in sleep timing and sleep duration by upwards of one hour, most of the existing adolescent intervention studies (which primarily focus on sleep hygiene) have shown marginal impacts on weeknight variability [59,73,74] or weekend sleep onset variability [73]. Of the few adolescent sleep timing interventions, they have at most moved bedtimes approximately one hour earlier [30,59]—compared to bed and wake times that moved two hours earlier in our study. To our knowledge, the FIRST Program has produced stronger effects on stabilizing and moving sleep earlier across multiple indices of sleep than any other pediatric sleep intervention to date. While these findings are limited by the small sample, we hope that they motivate further inquiry into the magnitude of IIPT intervention effects on sleep. Although an equivalent intervention may be unavailable to many patients, the malleability of sleep is an important message for youth with chronic pain and co-morbid sleep complaints, who may feel that they have little control over their sleep schedules. This study finds that it is possible for these youth to have a healthy and consistent sleep/wake schedule with consistent scheduling and a function-focused approach. Our study also found that it is ultimately likely to be difficult to sustain these improvements in the home environment. Future work in this area should begin to focus on the follow-up period after IIPT discharge and investigate how to assist patients in maintaining their hard-earned sleep gains once they return home.

## Figures and Tables

**Table 1:** Sample characteristics.

	FIRST Patients	Comparison Group	*p*
	(*N* = 10)	(*N* = 9)	
Age (years, mean ± SD)	15.39 (2.14)	16.24 (1.49)	0.33
Sex (% female)	8 (80%)	7 (78%)	0.91
Race (*N*, %)			0.28
Caucasian	10 (100%)	8 (89%)	
Bi-racial	0 (0%)	1 (11%)	
Primary pain location (*N*, %)			0.50
Widespread musculoskeletal pain	6 (60%)	5 (56%)	
Localized musculoskeletal pain	3 (30%)	1 (11%)	
Abdominal pain	0 (0%)	2 (22%)	
Headache	1 (10%)	1 (11%)	
Insurance (*N*, %)			0.94
Commercial	9 (90%)	8 (89%)	
Medicaid	1 (10%)	1 (11%)	
Disability (FDI, mean ± SD)			0.36
Time 1 (baseline) ^a^	35.10 (7.92)	30.38 (13.17)	
Season recruited (*N*, %)			0.86
Fall	1 (10%)	2 (22%)	
Winter	4 (40%)	3 (33%)	
Spring	3 (30%)	3 33%)	
Summer	2 (20%)	1 (11%)	
Visits Completed Pre-COVID-19 shutdown ^b^ (*N*, %)			
Time 1 (baseline)	10 (100%)	9 (100%)	-
Time 2 (final IIPT week or equiv. time)	7 (70%)	5 (56%)	0.52
Time 3 (follow up)	7 (70%)	3 (33%)	0.11
Structured School Attendance (*N*, %) ^c^			
Time 1 (baseline)	30%	33%	0.88
Time 2 (final IIPT week or equiv. time)	-	29%	-
Time 3 (follow up)	73%	18%	0.05
Number of days between visits ^d^ (mean, min–max)			
Time 1 to Time 2	40 (9–65)	59 (38–76)	0.04 *
Time 2 to Time 3	50 (26–80)	58 (50–80)	0.29
FIRST Program Length in days (mean, ± SD, min–max)			
	20 (5.28; 11–29)	-	-
Post-Study for Clinical Sleep Follow-Up Referral			
Behavioral Sleep Medicine ^e^	30%	22%	0.52
Sleep Medicine ^f^	30%	55%	

Notes: * indicates *p* < 0.05. IIPT = Intensive Interdisciplinary Pain Treatment; FIRST Patients = patients receiving IIPT through the Functional Independence Restoration (FIRST) Program; Comparison Group = similarly disabled sample receiving outpatient treatment as usual; FDI = Functional Disability Inventory. ^a^ Time points corresponded to before (baseline, Time 1), during (final IIPT week or equivalent time, Time 2), and following (post-discharge follow up or equivalent time, Time 3) inpatient IIPT. ^b^ Time points completed prior to 17 March 2020 (the date that in-person school attendance was shut down in the state of Ohio due to declaration of state of emergency) were coded as “pre-shutdown” and time points that were completed after 17 March 2020 were coded as “during shutdown”. ^c^ School attendance is presented as percentage attending structured school (excluding unstructured homeschooling or online school). No value for FIRST Patients at Time 2 because they were at IIPT and thus not in school. ^d^ Number of days between visits was computed as such: first day of Time 2—last day of Time 1, etc. ^e^ Referred to see a behavioral sleep medicine psychologist for such sleep issues as perceived poor quality of sleep, excessive daytime sleepiness or fatigue, or perceived long sleep onset latency. ^f^ Referred to see a sleep medicine specialist for sleep issues such as low sleep efficiency.

**Table 2:** Descriptive sleep outcomes by group across time points.

	FIRST Patients		Comparison Group		Mean Difference	*p*
	Mean (SD)	Min–Max	Mean (SD)	Min–Max		
**Sleep Timing: Onset**						
Average Timing (Clock Time)						
Time 1	12:17 a.m. (151)	9:59 p.m.–6:48 a.m.	11:35 p.m. (53)	10:12 p.m.–1:15 a.m.	−45 min	0.14
Time 2	10:26 p.m. (29)	9:23 p.m.–11:06 p.m.	1:05 a.m. (110)	11:30 p.m.–4:50 a.m.	160 min	0.004
Time 3	11:49 p.m. (139)	10:14 p.m.–5:42 a.m.	12:49 a.m. (142)	10:44 p.m.–6:05 a.m.	60 min	0.90
Intra-Individual Variability (min)						
Time 1	76 (33)	32–136	60 (29)	23–115	−16 min	0.62
Time 2	29 (15)	6–60	65 (43)	24–155	35 min	0.03
Time 3	50 (20)	18–78	95 (95)	14–303	45 min	0.01
**Sleep Timing: Offset**						
Average Timing (Clock Time)						
Time 1	9:04 a.m. (159)	6:28 a.m.–3:57 p.m.	8:34 a.m. (70)	6:28 a.m.–10:22 a.m.	−30 min	0.29
Time 2	7:16 a.m. (28)	6:42 a.m.–8:02 a.m.	9:19 a.m. (102)	7:30 a.m.–12:42 p.m.	122 min	0.01
Time 3	8:36 a.m. (156)	6:01 a.m.–2:52 p.m.	9:49 a.m. (117)	7:23 a.m.–1:07 p.m.	73 min	0.72
Intra-Individual Variability (min)						
Time 1	90 (62)	42–241	88 (51)	27–161	−2 min	0.99
Time 2	21 (5)	15–35	85 (43)	8–142	64 min	0.002
Time 3	73 (50)	17–164	86 (58)	22–218	13 min	0.94
**Sleep Period (Duration)**						
Average Period (Mean h:min)						
Time 1	8:49 (60)	7:05–10:16	9:00 (63)	8:06–10:57	11 min	0.88
Time 2	8:52 (43)	8:06–10:15	8:14 (53)	6:29–9:45	−38 min	0.82
Time 3	8:49 (59)	7:26–10:06	9:02 (89)	6:14–11:36	13 min	0.62
Intra-Individual Variability (min)						
Time 1	107 (65)	63–277	88 (52)	26–191	−19 min	0.88
Time 2	35 (17)	16–70	89 (37)	34–138	54 min	0.01
Time 3	76 (32)	37–125	84 (53)	32–186	8 min	0.12
**Sleep Quality**						
Average Efficiency (%)						
Time 1	87% (8)	74–96%	80% (8)	64–90%	−7%	0.23
Time 2	86% (8)	71–94%	83% (12)	56–95%	−3%	0.48
Time 3	86% (8)	74–96%	80% (12)	57–94%	−6%	0.23

Notes: FIRST Patients = patients receiving IIPT through the Functional Independence Restoration (FIRST) Program; Comparison Group = similarly disabled sample receiving outpatient treatment as usual. Outcomes were collected in patients across three time point corresponding to before (baseline, Time 1), during (final IIPT week or equivalent time, Time 2), and following (post-discharge follow up or equivalent time, Time 3) inpatient IIPT. Standard deviations (SD) always in minutes (except for sleep efficiency). Intra-individual variability represents the average of the within-person standard deviation across participants. This differs from standard deviation (SD) reported under each average sleep measure, which represents the standard deviation of average values across participants. Mean difference computed by taking Comparison Group value—FIRST Patient value.

**Table 3:** Mixed ANOVA results for sleep outcomes by group (*FIRST Patients* vs. *Comparison Group*) across time points.

	**Sleep Onset**				**Sleep Offset**				**Duration**				**Sleep Efficiency**			
	***df***	***F***	***p***	***Partial eta*^2^**	***df***	***F***	***p***	***Partial eta*^2^**	***df***	***F***	***p***	***Partial eta*^2^**	***df***	***F***	***p***	***Partial eta*^2^**
Between subjects																
Group	1	1.80	0.20	0.10	1	1.58	0.23	0.09	1	0.03	0.88	0.002	1	1.81	0.20	0.1
Within subjects																
Time	2	0.91	0.41	0.05	2	2.28	0.12	0.12	2	2.05	0.13	0.11	2	0.46	0.63	0.03
Time x Group	2	8.06	0.001 *	0.32	2	4.49	0.02 *	0.21	2	2.68	0.08	0.14	2	1.19	0.32	0.07
	**Sleep Onset Variability**				**Sleep Offset Variability**				**Duration Variability**				**Sleep Efficiency Variability**			
	***df***	***F***	***p***	***Partial eta*^2^**	***df***	***F***	***p***	***Partial eta*^2^**	***df***	***F***	***p***	***Partial eta*^2^**	***df***	***F***	***p***	***Partial eta*^2^**
Between subjects																
Group	1	1.59	0.23	0.09	1	1.87	0.19	0.1	1	0.82	0.38	0.05	1	4.72	0.04 *	0.217
Within subjects																
Time	2	3.00	0.06 *	0.15	2	5.65	0.01 *	0.25	2	4.14	0.03 *	0.20	2	0.43	0.65	0.03
Time x Group	2	4.53	0.02 *	0.21	2	4.69	0.02 *	0.22	2	4.53	0.02 *	0.21	2	2.49	0.10	0.13

Notes: * indicates *p* < 0.05. FIRST Patients = patients receiving IIPT through the Functional Independence Restoration (FIRST) Program; Comparison Group = similarly disabled sample receiving outpatient treatment as usual.

**Table 4:** Post-hoc follow ups comparing *FIRST Patients* to *Comparison Group* at Time 2 and Time 3, controlling for Time 1 (baseline).

	Time 2 (Controlling for Time 1)							Time 3 (Controlling for Time 1)						
	SS	*df*	MS	*F*	*p*	*Partial Eta* ^2^	R^2^	SS	*df*	MS	*F*	*p*	*Partial Eta* ^2^	R^2^
**Sleep Onset**														
T1 Sleep Onset	9848	1	9848	1.64	0.22	0.09		171422	1	171422	16.61	0.00 *	0.51	
Group	128769	1	128769	21.39	0.00 *	0.57	0.57	42219	1	42219	4.09	0.06	0.20	0.53
Error	96321	16	6020					165088	16	10318				
**Sleep Offset**														
T1 Sleep Offset	11543	1	11543	2.37	0.14	0.13		150445	1	150445	13.52	0.00 *	0.46	
Group	76883	1	76883	15.76	0.00 *	0.50	0.51	42727	1	42727	3.84	0.07	0.19	0.50
Error	78075	16	4880					178038	16	11127				
**Sleep Onset Variability**														
T1 Sleep Onset Var.	844	1	844	0.83	0.38	0.05		24672	1	24672	7.83	0.01 *	0.33	
Group	6634	1	6634	6.56	0.02	0.29	0.29	18757	1	18757	5.95	0.03	0.27	0.41
Error	16181	16	1011					50403	16	3150				
**Sleep Offset Variability**														
T1 Sleep Offset Var.	4584	1	4584	6.89	0.02 *	0.30		19176	1	19176	10.29	0.01 *	0.39	
Group	19412	1	19412	29.16	0.00 *	0.65	0.69	872	1	872	0.47	0.50	0.03	0.40
Error	10652	16	666					29816	16	1863				
**Duration Variability**														
T1 Sleep Dur Var.	2258	1	2258	3.19	0.09	0.17		1015	1	1015	0.53	0.48		
Group	15118	1	15118	21.38	0.00 *	0.57	0.58	437	1	437	0.23	0.64	0.01	0.04
Error	11312	16	707					30763	16	1923				

Note: * indicates *p* < 0.025. FIRST Patients = patients receiving IIPT through the Functional Independence Restoration (FIRST) Program; Comparison Group = similarly disabled sample receiving outpatient treatment as usual. T1 = Time 1 (baseline). Var. = variability (intra-individual standard deviation). SS = Sum of Squares. MS = Mean Squares.

## Data Availability

The data presented in this study are available upon request from the corresponding author. Given the study’s small sample, the data are not publicly available to protect the privacy of participants.

## Supplementary Materials

The following are available online at https://www.mdpi.com/2227-9067/8/1/42/s1, Table S1: Mixed ANOVA results for sleep outcomes, removing outliers, by group (FIRST Patients vs. Comparison Group) across time points. Table S2: Mixed ANOVA results for sleep outcomes, removing outliers and using log transformation, by group (FIRST Patients vs. Comparison Group) across time points. Table S3: Mixed ANOVA results for weeknight-only sleep outcomes by group (FIRST Patients vs. Comparison Group) across time points. Table S4: Mann-Whitney U Test results for post-hoc group differences in sleep outcomes, removing outliers, from Time 1 to Time 2 and Time 1 to Time 3.
