# Supplementary material for: Sleep among Youth with Severely Disabling Chronic Pain: Before, during, and after Inpatient Intensive Interdisciplinary Pain Treatment
Source: Children (Basel). 2021 Jan 12;8(1):42. doi: 10.3390/children8010042 (PMC7828171; doi:10.3390/children8010042)
Supplement: Supplementary file 1 [file children-08-00042-s001.pdf]

Table S1. Mixed ANOVA results for sleep outcomes, removing outliers, by group (*FIRST Patients* vs. *Comparison Group*) across timepoints.

	**Sleep Onset**<br>FIRST outliers removed: 1<br>CG outliers removed: 1				**Sleep Offset**<br>FIRST outliers removed: 1<br>CG outliers removed: 0				**Duration**<br>FIRST outliers removed: 2<br>CG outliers removed: 3				**Sleep Efficiency**<br>FIRST outliers removed: 1<br>CG outliers removed: 1			
	df	*F*	*p*	*Partial eta*^{2}	*df*	*F*	*p*	*Partial eta*^{2}	*df*	*F*	*p*	*Partial eta*^{2}	*df*	*F*	*p*	*Partial eta*^{2}
Between subjects																
Group	**1**	**8.29**	**.01**	**.36**	**1**	**7.68**	**.01**	**.32**	1	.002	.97	.000	1	1.68	.21	.10
Within subjects																
Time	2	.21	.81	.01	2	1.64	.21	.09	2	2.58	.10	.18	2	1.16	.33	.07
Time x Group	**2**	**7.34**	**.003**	**.33**	**2**	**4.28**	**.02**	**.21**	2	3.17	.06	.21	2	1.73	.20	.10

	**Sleep Onset Variability**<br>FIRST outliers removed: 0<br>CG outliers removed: 1				**Sleep Offset Variability**<br>FIRST outliers removed: 0<br>CG outliers removed: 1				**Duration Variability**<br>FIRST outliers removed: 1<br>CG outliers removed: 0			
	df	*F*	*p*	*Partial eta*^{2}	*df*	*F*	*p*	*Partial eta*^{2}	*df*	*F*	*p*	*Partial eta*^{2}
Between subjects												
Group	1	.46	.51	.03	1	.90	.36	.05	1	.27	.61	.02
Within subjects												
Time	2	3.12	.06	.16	**2**	**5.0**	**.01**	**.24**	**2**	**5.18**	**.01**	**.25**
Time x Group	**2**	**5.48**	**.01**	**.26**	**2**	**7.36**	**.002**	**.32**	**2**	**4.18**	**.02**	**.21**

Notes: * indicates p<.05. FIRST Patients = patients receiving IIPT through the Functional Independence Restoration (FIRST) Program; Comparison Group = similarly disabled sample receiving outpatient treatment-as-usual.

Table S2. Mixed ANOVA results for sleep outcomes, removing outliers and using log transformation, by group (*FIRST Patients* vs. *Comparison Group*) across timepoints.

	**Sleep Onset** FIRST outliers removed: 1 CG outliers removed: 1				**Sleep Offset** FIRST outliers removed: 1 CG outliers removed: 0				**Duration** FIRST outliers removed: 2 CG outliers removed: 3				**Sleep Efficiency** FIRST outliers removed: 1 CG outliers removed: 1			
	df	*F*	*p*	*Partial eta²*	*df*	*F*	*p*	*Partial eta²*	*df*	*F*	*p*	*Partial eta²*	*df*	*F*	*p*	*Partial eta²*
Between subjects																
Group	**1**	**8.74**	**.01***	**.37**	**1**	**7.8**	**.01***	**.33**	1	.000	1.00	.000	1	1.65	.22	.10
Within subjects																
Time	2	.21	.81	.01	2	1.60	.22	.09	2	2.36	.12	.16	2	1.32	.28	.08
Time x Group	2	7.5	.002*	.33	**2**	**4.35**	**.02***	**.21**	2	3.31	.05	.22	2	1.68	.20	.10

	**Sleep Onset Variability** FIRST outliers removed: 0 CG outliers removed: 1				**Sleep Offset Variability** FIRST outliers removed: 0 CG outliers removed: 1				**Duration Variability** FIRST outliers removed: 1 CG outliers removed: 0			
	df	*F*	*p*	*Partial eta²*	*df*	*F*	*p*	*Partial eta²*	*df*	*F*	*p*	*Partial eta²*
Between subjects												
Group	1	.60	.45	.04	1	1.98	.18	.11	1	3.16	.10	.17
Within subjects												
Time	2	4.60	.02*	.22	**2**	**14.91**	**.000***	**.48**	**2**	**5.84**	**.01***	**.27**
Time x Group	**2**	**4.90**	**.01***	**.23**	**2**	**14.77**	**.000***	**.48**	**2**	**9.12**	**.001***	**.36**

Notes: * indicates p<.05. FIRST Patients = patients receiving IIPT through the Functional Independence Restoration (FIRST) Program; Comparison Group = similarly disabled sample receiving outpatient treatment-as-usual.

Table S3. Mixed ANOVA results for <u>weeknight only</u> sleep outcomes by group (*FIRST Patients* vs. *Comparison Group*) across timepoints.

	Sleep Onset				Sleep Offset				Duration				Sleep Efficiency			
	df	*F*	*p*	*Partial eta*^{2}	*df*	*F*	*p*	*Partial eta*^{2}	*df*	*F*	*p*	*Partial eta*^{2}	*df*	*F*	*p*	*Partial eta*^{2}
Between subjects																
Group	1	1.26	.28	.07	1	1.99	.18	.11	1	.11	.74	.01	1	1.81	.20	.10
Within subjects																
Time	2	.62	.54	.04	2	1.97	.16	.10	2	2.93	.07	.15	2	.14	.87	.01
Time x Group	**2**	**4.68**	**.02**	**.22**	2	2.54	.09	.13	**2**	**3.38**	**.05**	**.17**	2	.18	.84	.01
	**Sleep Onset Variability**				**Sleep Offset Variability**				**Duration Variability**							
	df	*F*	*p*	*Partial eta*^{2}	*df*	*F*	*p*	*Partial eta*^{2}	*df*	*F*	*p*	*Partial eta*^{2}				
Between subjects																
Group	1	1.10	.31	.06	1	1.07	.32	.06	1	1.16	.30	.06				
Within subjects																
Time	2	1.54	.23	.08	2	3.02	.06	.15	**2**	**3.24**	**.05**	**.16**				
Time x Group	**2**	**4.38**	**.02**	**.21**	**2**	**3.42**	**.05**	**.17**	2	2.09	.14	.11				

Notes: * indicates p<.05. FIRST Patients = patients receiving IIPT through the Functional Independence Restoration (FIRST) Program; Comparison Group = similarly disabled sample receiving outpatient treatment-as-usual.

Table S4. Post-hoc group differences in sleep outcomes, removing outliers, from Time 1 to Time 2, and Time 1 to Time 3 via the Mann-Whitney U Test

	FIRST Patients	Comparison Group		
	**Mean Rank**	**Mean Rank**	**Z-value**	**p-value**
**Sleep Onset**	N = 9	N = 8		
Time 2 – Time 1	5.67	12.75	-2.89	.002*
Time 3 – Time 1	7.22	11.00	-1.54	.14
**Sleep Offset**	N = 9	N = 9		
Time 2 – Time 1	6.33	12.67	-2.52	.01*
Time 3 – Time 1	8.00	11.00	-1.19	.26
**Sleep Onset Variability**	N = 10	N = 8		
Time 2 – Time 1	6.70	13.00	-2.49	.01*
Time 3 – Time 1	7.50	12.00	-1.78	.08
**Sleep Offset Variability**	N = 10	N = 8		
Time 2 – Time 1	14.13	10.13	-3.29	.000*
Time 3 – Time 1	5.80	9.00	-.44	.67
**Duration Variability**	N = 9	N = 9		
Time 2 – Time 1	6.89	12.11	-2.08	.04
Time 3 – Time 1	9.00	10.00	-.40	.73

Notes: * indicates $p \leq .01$. FIRST Patients = patients receiving IIPT through the Functional Independence Restoration (FIRST) Program; Comparison Group = similarly disabled sample receiving outpatient treatment-as-usual.
